# Novel Galiellalactone Analogues Can Target STAT3 Phosphorylation and Cause Apoptosis in Triple-Negative Breast Cancer

**DOI:** 10.3390/biom9050170

**Published:** 2019-05-03

**Authors:** Hyejin Ko, Jong Hyun Lee, Hyun Su Kim, Taewoo Kim, Young Taek Han, Young-Ger Suh, Jaemoo Chun, Yeong Shik Kim, Kwang Seok Ahn

**Affiliations:** 1 Natural Products Research Institute, College of Pharmacy, Seoul National University, 1 Gwanak-ro, Gwanak-gu, Seoul 08826, Korea; hyejinko@snu.ac.kr (H.K.); jaemoo.chun@gmail.com (J.C.); 2 Department of Science in Korean Medicine, Kyung Hee University, 24 Kyungheedae-ro, Dongdaemun-gu, Seoul 02447, Korea; jhlee0801@khu.ac.kr; 3 College of Pharmacy, CHA University, 120 Haeryong-ro, Pochen-si, Gyenggi-do 11160, Korea; khs8812@snu.ac.kr (H.S.K.); ygsuh@cha.ac.kr (Y.G.S.); 4 College of Pharmacy, Seoul National University, 1 Gwanak-ro, Gwanak-gu, Seoul 08826, Korea; taewookim@snu.ac.kr; 5 College of Pharmacy, Dankook University, 119 Dandae-ro, Dongnam-gu, Cheonan 330-714, Korea; hanyt@dankook.ac.kr

**Keywords:** STAT3, galiellalactone, triple-negative breast cancers (TNBCs), apoptosis, radiation, xenograft

## Abstract

Aberrant activation of signal transducer and activator of transcription 3 (STAT3) has been documented in various malignancies including triple-negative breast cancers (TNBCs). The STAT3 transcription factor can regulate the different important hallmarks of tumor cells, and thus, targeting it can be a potential strategy for treating TNBC, for which only limited therapeutic options are available. In this study, we analyzed the possible effect of (-)-galiellalactone and its novel analogues, SG-1709 and SG-1721, and determined whether these agents exerted their antineoplastic effects by suppressing the STAT3 signaling pathway in TNBC cells. The two analogues, SG-1709 and SG-1721, inhibited both constitutive as well as inducible STAT3 phosphorylation at tyrosine 705 more effectively than (-)-galiellalactone, which indicates that the analogues are more potent STAT3 blockers. Moreover, SG-1721 not only inhibited nuclear translocation and DNA binding of STAT3 but also induced apoptosis, and decreased expression of diverse oncogenic proteins. Interestingly, SG-1721 also exhibited an enhanced apoptotic effect when combined with radiotherapy. Furthermore, in vivo administration of SG-1721 significantly attenuated breast xenograft tumor growth via decreasing levels of p-STAT3. Therefore, SG-1721 may be a promising candidate for further application as a pharmacological agent that can target STAT3 protein in treating TNBC.

## 1. Introduction

Breast cancer is the most commonly diagnosed cancer among women worldwide [1,2,3,4,5,6]. There were over two million new cases in 2018 [7,8,9,10,11,12,13,14,15] and more than 40,000 deaths due to breast cancer each year in the United States [16]. Clinically, breast cancer can be divided into four distinct subtypes, namely: Luminal A, luminal B, human epidermal growth factor receptor 2 (HER2) overexpression, and basal-like (Basal A and Basal B) [17]. However, breast cancer is still generally assessed based on the expression of estrogen receptor (ER), progesterone receptor (PR), and HER2 [18]. Triple-negative breast cancer (TNBC) does not express these three molecular markers but exhibits a more aggressive phenotype and has a high risk of recurrence, with mortality ranging from 3 to 5 years after diagnosis with limited treatment options [19]. Therefore, there is a major need to understand the molecular basis of TNBC for the development of effective therapeutic strategies for patients with TNBC. There are many oncogenic molecules that are involved in the progression of breast cancer, and signal transducer and activator of transcription 3 (STAT3) protein can play a multifaceted role in breast tumorigenesis [20,21,22,23,24,25].

STAT3 protein has been shown to be constitutively active in a large number of tumor tissues, including those derived from breast cancer patients [25,26,27,28,29,30,31,32,33,34,35,36,37]. STAT3 can be induced by the leukemia inhibitory factor in normal breast cells, whereas STAT3 is activated by IL-6 stimulation in breast cancer [38]. In addition, intrinsic kinases such as EGFR (epidermal growth factor receptor) and VEGFR (vascular endothelial growth factor receptor) can mediate aberrant phosphorylation of STAT3 in breast cancer [39]. Upon its activation, this transcription factor can regulate proliferation, survival, and metastasis in TNBC and also lead to resistance to chemotherapy as well as radiotherapy [20,21,22,23,24,25]. Several studies also described that STAT3 was activated in TNBC, suggesting that therapeutic inhibition of STAT3 signaling might be effective in TNBC [40,41]. Therefore, finding a potent small molecule which could significantly inhibit STAT3 activation is required.

Galiellalactone (GL), a STAT3 pharmacological inhibitor, is a hexaketide metabolite that can selectively block the binding of active STAT3 dimers to DNA [42]. In spite of tremendous progress in discovery of new synthetic drugs for cancer treatment, application of natural products for targeting aberrant growth and metastasis of tumor cells remains an important therapeutic strategy [43,44,45,46,47]. Moreover, as per the recent reports, GL can significantly attenuate the growth of prostate cancer cells and cause apoptosis [48]. It also inhibits DNA binding of the Smad2/3 transcription factor in both HepG2 as well as MDA–MB-231 cells and also antagonizes the cellular effects of transforming growth factor-beta (TGF-β) [49]. A few prior studies related to the structure–activity relationship of GL have been predominantly performed focused on modifying the basic hydrindane system of GL [50]. Recently, we identified a series of novel STAT3-selective inhibitors based on GL via the truncation of the cyclohexene ring system [51]. Among the structural analogues, SG-1709 and SG-1721 exhibited potent cytotoxicity against TNBC cell lines via the specific inhibitory activity of the phosphorylated STAT3 at the tyrosine 705 residue and selectively inhibited the STAT3 signaling pathway without suppressing the levels of STAT1 and STAT5.

In this study, we examined whether the cyclohexene-truncated bicyclic lactone analogues SG-1709 and SG-1721 derived from natural GL can inhibit tumor growth by effectively blocking the STAT3 signaling pathway in vitro and in vivo in breast cancers. We also investigated whether exposure of GL analogue in conjunction with ionizing radiation can augment apoptosis compared to individual treatment in breast cancer cells.

## 2. Materials and Methods

### 2.1. Reagents

(-)-Galiellalactone and its analogues SG-1709, or SG-1721 were dissolved in DMSO, and the final concentration of DMSO in the cell culture was kept below 0.05%. Dulbecco’s modified eagle medium (DMEM), RPMI 1640 medium, fetal bovine serum (FBS), antibiotic–antimycotic mixture, and LightShift^®^ Chemiluminescent EMSA kit were obtained from Thermo Fisher Scientific Inc. (Waltham, MA, USA). 5′-biotinylated STAT3 was obtained from Bioneer Corporation (Daejeon, Korea). 3-(4,5-Dimethylthiazol-2-yl)-2,5-diphenyltetrazolium bromide (MTT) was purchased from Sigma Aldrich (St. Louis, MO, USA). Epidermal growth factor (EGF) and IL-6 were purchased from R&D Systems (Minneapolis, MN, USA). Antibodies against STAT3, Lamin B, Bcl-xL, Bcl-2, Cyclin D1, MMP-9, PARP (Poly (ADP-ribose) polymerase), Caspase-3, MMP-2, COX-2, Ki-67, and β-actin were purchased from Santa Cruz Biotechnology (Santa Cruz, CA, USA); p-STAT3(Tyr705), p-JAK1(Tyr1022/1023), JAK1, p-JAK2 (Tyr1007/1008), JAK2, Cleaved caspase-3, and Cyclin D1 were purchased from Cell signaling Technology (Beverly, MA, USA). 

### 2.2. Cell Lines

Human triple-negative breast cancer cell lines (BT-549, BT-20, and MDA–MB-468), luminal A breast cancer cell lines (MCF-7 and T47D), HER2 breast cancer cell lines (SK-BR-3 and MDA–MB-453), and nontumorigenic epithelial cell lines MCF-10A were purchased from the American Type Culture Collection (Manassas, VA, USA). The MDA–MB-468 and MDA–MB-453 cells were grown in DMEM supplemented with 10% FBS and 1% antibiotics (penicillin 100 U/mL and streptomycin 100 μg/mL). 

### 2.3. Western Blot Assay

MDA–MB-468 cells were seeded in 6-well plates at a density of 1 × 10^6^ cells/well. The cells were treated with the indicated concentrations (5 or 10 μM) of (-)-galiellalactone (GL), its active analogues SG-1709, or SG-1721 for 4 or 24 h. Whole cell lysates were prepared using a lysis buffer (20 mM HEPES, pH 7.6, 350 mM NaCl, 20% glycerol, 0.5 mM EDTA, 0.1 mM EGTA, 1% NP-40, 50 mM NaF, 1 mM DTT, 1 mM PMSF, protease inhibitor cocktail, and phosphatase inhibitor cocktail). The lysates were centrifuged, and the supernatant was collected. Equal amounts of protein were resolved on 8-15% SDS-PAGE and transferred to a nitrocellulose membrane. The membrane was blocked with 5% bovine serum albumin (BSA) and incubated with primary antibodies overnight at 4 °C. The blots were washed and incubated with secondary antibodies conjugated with HRP (horseradish peroxidase) for 1 h and then examined by chemiluminescence (Intron Biotechnology, Seoul, Korea) and visualized using a LAS-1000 image analyzer (Fujifilm, Tokyo, Japan).

### 2.4. Immunocytochemistry for p-STAT3 and STAT3 Localization

The MDA–MB-468 cells were plated on coverslips and allowed to attach by overnight incubation. The cells were treated with 10 μM of GL, SG-1709, or SG-1721 for 4 h and fixed with 4% paraformaldehyde (PFA) for 20 min at room temperature. The cells were permeabilized with 0.2% Triton X-100 in PBS (phosphate buffered saline) for 20 min. After a brief washing in PBS, slides were blocked with 5% bovine serum albumin (BSA) for 1 h at room temperature, then incubated either with goat polyclonal anti human p-STAT3 antibody (dilution, 1:100) or with rabbit polyclonal anti human STAT3 antibody (dilution, 1:100) overnight at 4 °C. The slides were washed and then incubated either with Alexa Fluor 488 (dilution, 1:1000) antigoat IgG1 or with Alexa Fluor 594 (dilution, 1:1000) antirabbit IgG1 for 1 h at room temperature in the dark. Next, counterstained for nuclei with 5 μg/mL DAPI (4’,6-diamidino-2-phenylindole) solution for 3 min. Stained slides were mounted with mounting medium (GBI Laboratories, Manchester, UK) and analyzed under an Olympus FluoView FV1000 confocal microscope (Tokyo, Japan). DAPI and FITC (fluorescein-5-isothiocyanate) fluorescence were excited (Ex: 405 nm and 488 nm) and detected (Em: 461 nm and 519 nm) with 2.1% laser transmissivity and 5.0% laser transmissivity, respectively.

### 2.5. Electrophoretic Mobility Shift Assay (EMSA) for STAT3-DNA Binding

STAT3-DNA binding was analyzed by an electrophoretic mobility shift assay (EMSA) using a 5′-biotinylated STAT3 oligonucleotide (5′-GATCCTTCTGGGAATTCCTAGATC-3′ and 5′-GATCTAGGAATTCCCAGAAGGATC-3′). Briefly, nuclear extracts were prepared from GL, SG-1709, or SG-1721-treated cells and incubated with the 5′-biotinylated STAT3 oligonucleotide probes. The DNA–protein complex formed was separated from free oligonucleotide on 6% native polyacrylamide gels and transferred to a positively charged nylon membrane. The membrane was detected following the manufacturer’s instructions using LightShift® Chemiluminescent EMSA kit (Waltham, MA, USA).

### 2.6. STAT3-Dependent Luciferase Reporter Assay

MDA–MB-468 cells were plated in 24-well plates at a density of 1×10^5^ cells/well. After 24 h, the cells were transiently transfected with a p-STAT3-Luc reporter vector in the presence of a pCMV-Luc vector (Firefly) using a transfection reagent (Intron Biotechnology, Seoul, Korea). At 24 h post-transfection, the cells were pretreated with 5 μM of GL, SG-1709, or SG-1721 for 24 h and then induced by IL-6 or EGF for additional 5 or 30 min. The luciferase assay was performed using the Dual Luciferase Reporter Assay System (Promega, Madison, WI, USA) in accordance with the manufacturer’s instructions. The luminescence signal was measured using a luminometer (MicroLumat Plus, Berthold Technologies, Dortmund, Germany). 

### 2.7. si-RNA Trnasfection

To test whether our drugs are really functioning through STAT3, transient transfections were carried out using STAT3 (Santa Cruz, CA, USA) and scrambled si-RNAs (Bioneer Corporation, Daejeon, Korea). MDA–MB-468 cells were plated in 24-well plates at a density 1 × 10^5^ cells/well. After 24 h, the cells were transiently transfected with STAT3 and scrambled (control) si-RNAs using a transfection reagent (Intron Biotechnology, Seoul, Korea). At 24 h post-transfection, the cells were treated with 10 μM of SG-1721 for 4 h or 24 h and then performed Western blot assay or annexin V assays.

### 2.8. Cell Cycle Analysis

To determine apoptosis, cell cycle analysis was performed using propidium iodide. MDA–MB-468 cells were plated in 6-well plates at a density of 1 × 10^6^ cells/well. After treatment with 10 μM of SG-1721 for 24 h, the cells were collected and washed with 1× PBS. Cell pellets were fixed in 70% cold ethanol overnight at −20 °C. The fixed cells were resuspended in 1× PBS containing 1 mg/mL RNase A, incubated for 1 h at 37 °C incubation. Cells were then washed, resuspended, and stained in PBS containing 25 μg/mL of propidium iodide for 30 min at room temperature in the dark. The DNA contents of the stained cells were analyzed using Cell Quest Software with a FACScan Calibur flowcytometry (BD Biosciences, Becton-Dickinson, Franklin Lakes, NJ, USA).

### 2.9. Annexin V Assays

MDA–MB-468 cells were seeded in plat and treated with 10 μM of SG-1721 for 24 h. Apoptosis was evaluated by annexin V-FITC and Propidium Iodide stained cells using FITC Annexin V Apoptosis Detection Kit I in accordance with the manufacturer’s protocols. Briefly, the cells were harvested using 1% trypsin in PBS (welgene, Gyeongsangbuk-do, Korea), washed once with cold PBS. The cell pellet was resuspended in 1× binding buffer add 5 μL of FITC Annexin V and 5 μL of propidium iodide (PI) for 15 min at room temperature in the dark. Stained samples were analyzed by FACScan Calibur flowcytometry (BD Biosciences, Becton-Dickinson, Franklin Lakes, NJ, USA).

### 2.10. TUNEL Assays

Individual apoptotic cell death was observed using a Roche Diagnosis TUNEL (Terminal transferase mediated dUTP-fluorescein nick end labeling) assay kit in accordance with the manufacturer’s instructions. Briefly, cells treated with 10 μM of SG-1721 for 24 h were washed with cold PBS. The cells were fixed with 4% paraformaldehyde for 30 min and washed twice with PBS. The resuspended cells were put in a permeabilization solution (0.1% Triton X-100 and 0.1% Sodium citrate) for 20 min at 4 °C, and then the cells were washed with cold PBS. The cells were subsequently incubated with a TUNEL enzyme and TUNEL label mixture for 1 h at 37 °C in a humidified atmosphere in the dark. After being washed with PBS, cells were analyzed using FACScan Calibur flowcytometry (BD Biosciences, Becton-Dickinson, Franklin Lakes, NJ, USA).

### 2.11. Cytotoxicity and Combination Index

To measure cytotoxicity, MTT assay was carried out as previously described [52]. To analyze the combination index, the effects of drug combinations were evaluated with Calcusyn software (Biosoft, Cambridge, UK), which employs the Chou–Talalay combination index method, which is based on the median–effect equation, itself a derivation from the mass–action law [53]. 

### 2.12. Irradiation and Cytotoxicity Assay

To measure cytotoxicity, MTT assay was carried out as previously described [52]. To analyze the combination index, the effects of drug combinations were evaluated with Calcusyn software (Biosoft, Cambridge, UK). This software uses the Chou–Talalay combination index method, which is based on the median–effect equation, itself a derivation from the mass–action law [53]. We entered the resulting data, along with the data obtained from single drug treatments, into Calcusyn to determine a combination index value (CI) for each combination point, which quantitatively defines additivity (CI = 1), synergy (CI < 1), and antagonism (CI > 1).

### 2.13. Animals

All procedures involving animals were reviewed and approved by Seoul National University Institutional Animal Care and Use committee (SNU-160908-1). Six-week-old athymic nu/nu female mice were purchased form Nara Biotec CO (Gyeonggi-do, Korea).

### 2.14. In Vivo Studies

When tumors had reached 0.25 cm in diameter, the mice were randomized into the following treatment groups (*n* = 6/group) based on the tumor volume. Group I (control) was treated with PBS and group II was treated with SG-1721 (0.5 mg/kg). Treatment was continued for up to 20 days from the date of randomization (Day 0). The tumors were carefully excised and measured as carried out previously [37]. 

### 2.15. Immunohistochemical Analysis of Tumor Samples

Solid tumors from control and treatment groups were fixed with 10% neutral buffered formalin (BBC Biochemical, USA), processed and embedded in paraffin. Sections were cut and deparaffinized in xylene and dehydrated in graded alcohol and finally hydrated in water. Antigen retrieval was performed by boiling the slide in 10 mM sodium citrate (pH 6.0) for 30 min. Immunohistochemistry was performed following manufacturer instructions (Vector Laboratories ImmPRESSTM REAGENT KIT). Briefly, endogenous peroxidases were quenched with 3% hydrogen peroxide. Nonspecific binding was blocked by incubation in the blocking reagent in the ImmPRESSTM REAGENT KIT (Vector Laboratories, Burlingame, CA, USA) in accordance with the manufacturer’s instructions. Sections were incubated overnight with primary antibody: p-STAT3, anti-Ki-67, and anticleaved caspase-3 (at 1:100 dilutions). Slides were subsequently washed several times in PBS and were incubated with ImmPRESSTM reagent in accordance with the manufacturer’s instructions. Immunoreactive species were detected using 3, 3-diaminobenzidine tetrahydrochloride (DAB) as a substrate. Sections were counterstained with Gill’s hematoxylin and mounted under glass cover slips. Images were taken using a Nikon Eclipse Ts2 inverted routine microscope (magnification, 40×).

### 2.16. Statistical Analysis

All the data are presented as the mean ± standard deviation (SD) from at least three independent experiments. An analysis of variance (ANOVA) with the Dunnett’s t-test was used for the statistical analysis of multiple comparisons. A value of *p* < 0.05 was chosen as the criterion for statistical significance.

## 3. Results

### 3.1. GL and Its Analogues Block Constitutive Phosphorylation of STAT3 in TNBC Cells 

**Figure 1:**
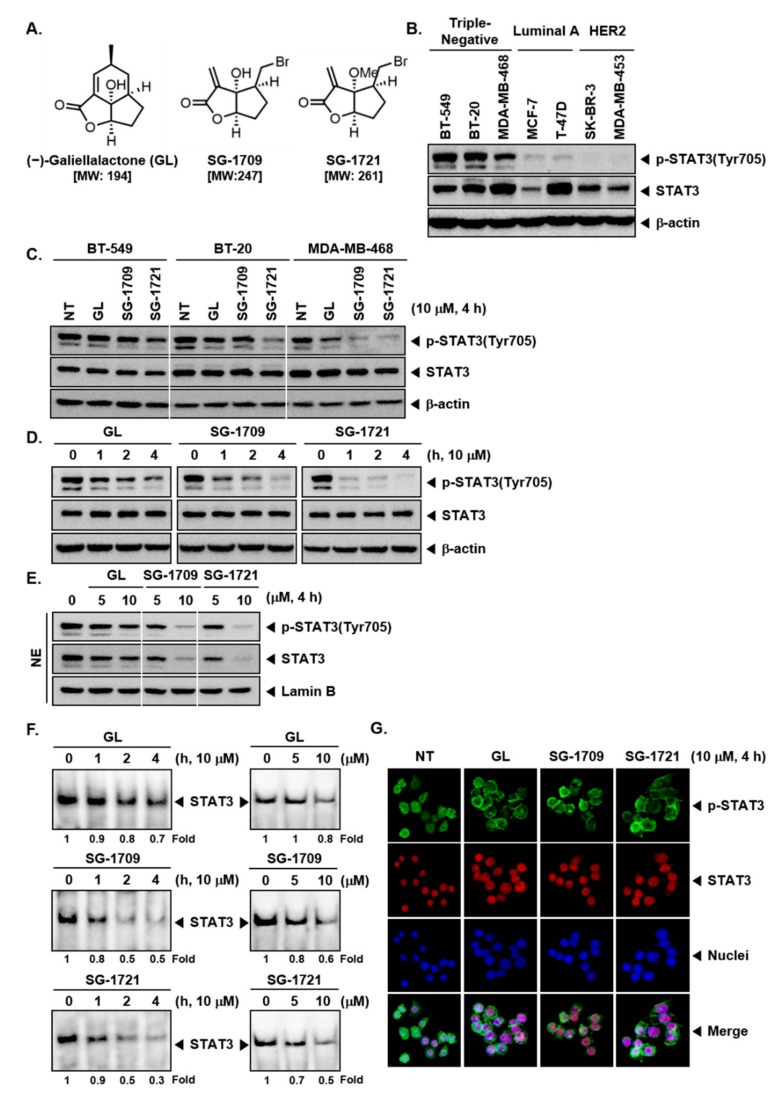
(-)-Galiellalactone (GL) and its analogues can block the STAT3 signaling pathway. (**A**) The chemical structure of (-)-galiellalactone (GL), SG-1709, and SG-1721. (**B**) Detection levels of p-STAT3 and STAT3 in seven human breast cancer cell lines were analyzed by Western blot analysis. (**C**) BT-549, BT-20, and MDA–MB-468 cells were treated with 10 μM of GL, SG-1709, and SG-1721 for 4 h, and Western blot analysis was done for different proteins. (**D**) MDA–MB-468 cells as described above for various time intervals and Western blot analysis was done. (**E**) MDA–MB-468 cells were treated with concentrations of GL, SG-1709, and SG-1721 for 4 h, and Western blot analysis was done. (**F**) MDA–MB-468 cells were treated as described above in panel D and E, and electrophoretic mobility shift assay was done. (**G**) MDA–MB-468 cells were treated with 10 μM of GL, SG-1709, and SG-1721 for 4 h, and immunocytochemistry was done.

The chemical structures of GL and its analogues, SG-1709 and SG-1721, are shown in Figure 1A. Firstly, we examined the phosphorylation status of STAT3 in different subtypes of breast cancer cells. We observed that STAT3 was persistently phosphorylated in TNBC cells BT-549, BT-20, and MDA–MB-468 cells. By contrast, STAT3 was expressed at very low levels in luminal A (MCF-7 and T-47D) and HER2 (SK-BR-3 and MDA–MB-453) breast cancer cells (Figure 1B). We next elucidated whether GL and its novel analogues can modulate STAT3 activation, on TNBC cells. It was found that the two analogues, SG-1709 and SG-1721, inhibited both constitutive and inducible STAT3 phosphorylation at tyrosine 705 more effectively than GL (Figure 1C). Moreover, as shown in Figure 1D, STAT3 phosphorylation was suppressed in MDA–MB-468 cells in a time-dependent manner, and SG-1721 most effectively inhibited STAT3 phosphorylation. 

### 3.2. GL and Its Analogues Act at Multiple Steps in STAT3 Signaling Pathway

It was deciphered next whether GL, SG-1709, and SG-1721 could also affect the nuclear translocation of STAT3. It was noted that 5 μM of GL, SG-1709, and SG-1721 slightly suppressed the translocation of STAT3 to the nuclei, but 10 μM of SG-1709 and SG-1721 inhibited this translocation of STAT3 into the nuclei more potently as compared to GL (Figure 1E). Next, it was confirmed by electrophoretic mobility shift assay (EMSA) that GL, SG-1709, and SG-1721 decreased STAT3–DNA binding activity in a time- and concentration-dependent manner, among which SG-1721 was most effective (Figure 1F). Furthermore, using immunocytochemistry, it was demonstrated that GL, SG-1709, and SG-1721 reduced the translocation of p-STAT3 and STAT3 to the nucleus in MDA–MB-468 cells (Figure 1G).

### 3.3. GL And Its Analogues Inhibit Inducible STAT3 Phosphorylation

**Figure 2:**
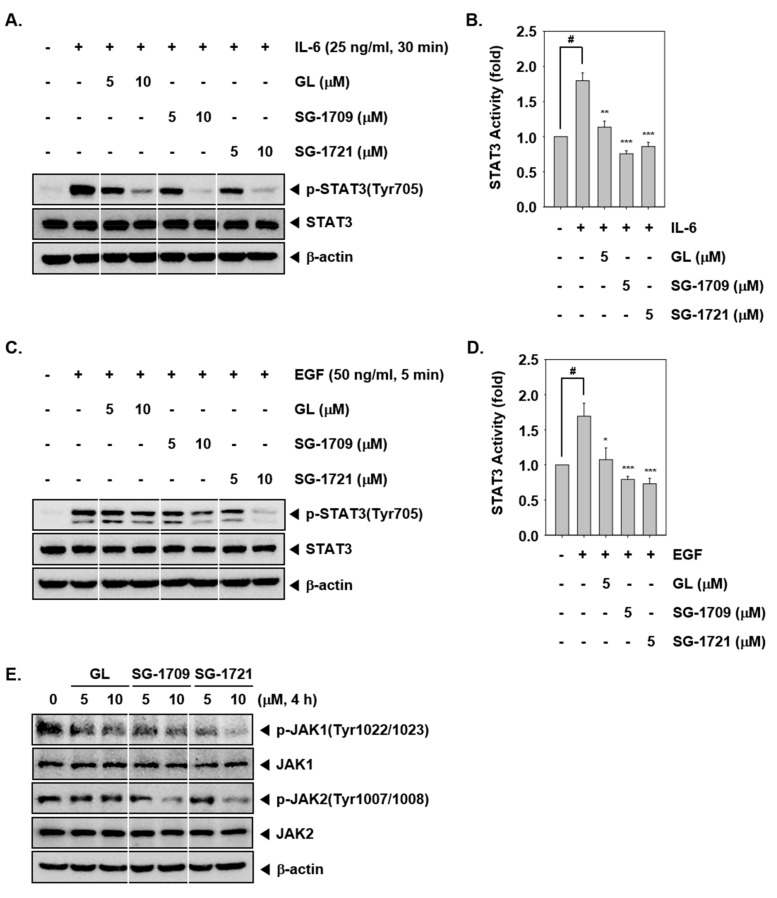
GL and its analogues inhibit inducible STAT3 activation. (**A**) MDA–MB-468 cells were pretreated with concentrations of GL, SG-1709, and SG-1721 for 4 h, then simulated with IL-6. Thereafter, equal amounts of lysates were analyzed by Western blot analysis using antibodies against p-STAT3 (Tyr705) and STAT3. (**B**) MDA–MB-468 cells were treated as described above in panel A, and luciferase activity was measured. The results shown are representative of three independent experiments. # *p* < 0.001; * *p* < 0.05, ** *p* < 0.01; *** *p* < 0.001. (**C**) MDA–MB-468 cells were pretreated as described above and then simulated with epidermal growth factor (EGF). Thereafter, Western blot analysis was carried out. (**D**) MDA–MB-468 cells were treated as described above in panel C, and luciferase activity was measured. The results shown are representative of three independent experiments. # *p* < 0.001; * *p* < 0.05, *** *p* < 0.001. (**E**) MDA–MB-468 cells were treated with concentrations of GL, SG-1709, and SG-1721 for 4 h, and Western blot analysis was done.

We also examined whether GL, SG-1709, and SG-1721 can also abrogate IL-6 or EGF-induced STAT3 phosphorylation in MDA–MB-468 cells. Results show that IL-6 (25 ng/mL) or EGF (50 ng/mL) caused substantial STAT3 activation and GL, SG-1709, and SG-1721 exposure inhibited inducible STAT3 phosphorylation. (Figure 2A,C). We also noted that upon pretreatment with GL, SG-1709, and SG-1721, IL-6 or EGF-induced STAT3 luciferase activity was inhibited and the two analogues were more effective than GL (Figure 2B,D).

### 3.4. GL and Its Analogues Reduce JAK1/2 Phosphorylation in MDA–MB-468 Cells

We next analyzed the effects of GL, SG-1709, and SG-1721 on JAK1 and JAK2 activation. As shown in Figure 2E, JAK1 and JAK2 were constitutively phosphorylated in MDA–MB-468 cells. Although treatment with 5 μM of GL, SG-1709, and SG-1721 did not completely block constitutive JAK1/2 activation 10 μM of SG-1709 and SG-1721 inhibited these activation more potently than GL. However, GL could only weakly suppress JAK1 and JAK2 phosphorylation.

### 3.5. GL and Its Analogues Suppress Cell Viability and Proliferation 

**Figure 3:**
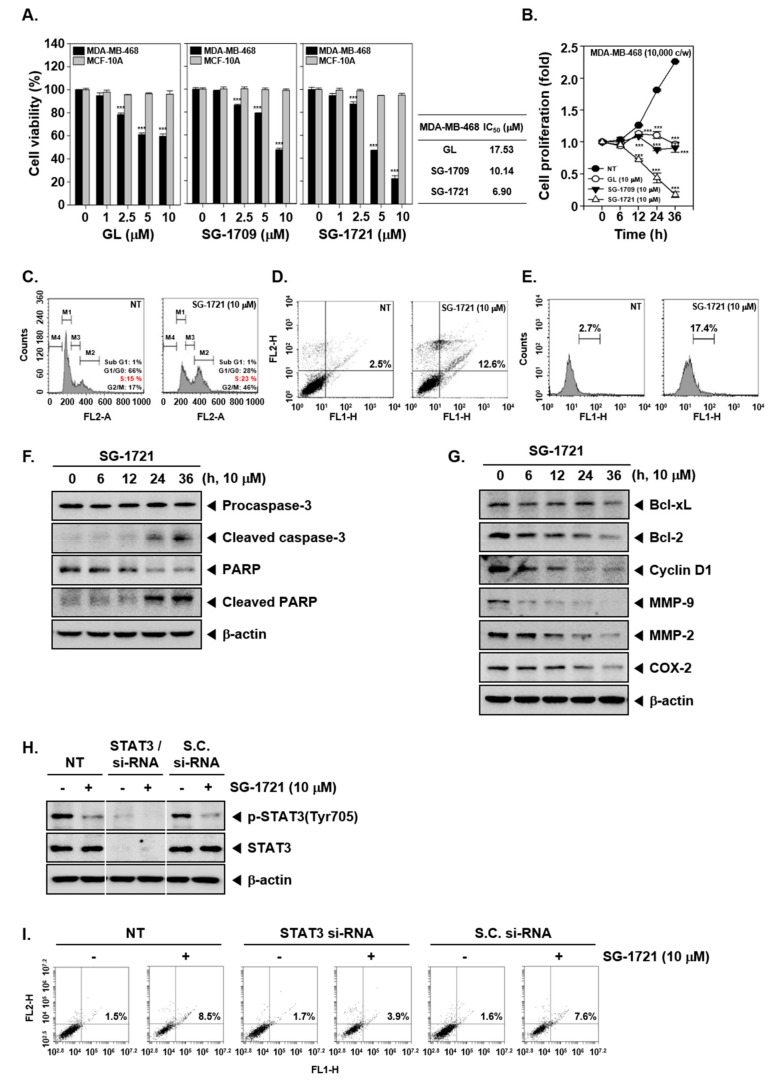
GL and its analogues can cause apoptosis. (**A**) MDA–MB-468 and MCF-10A cells were treated with various concentrations of GL, SG-1709, and SG-1721 for 24 h, and cell viability was determined using the MTT (3-(4,5-Dimethylthiazol-2-yl)-2,5-diphenyltetrazolium bromide) assay (**left panel**). IC_50_ values were determined as the drug concentration at 50% inhibition of the cell viability (**right panel**). *** *p* < 0.001. (**B**) MDA–MB-468 cells (10,000 cells/wells) were seeded for overnight and then treated with 10 μM of GL, SG-1709, and SG-1721, and cell proliferation assay was done. *** *p* < 0.001. (**C**) MDA–MB-468 cells were treated with 10 μM of SG-1721 for 24 h. Cellular DNA staining incorporating propidium iodide (PI) and flow cytometry analysis was carried out to determine the cell-cycle distribution. (**D**) MDA–MB-468 cells were treated as described in panel C, stained with FITC (fluorescein-5-isothiocyanate)-conjugated anti-Annexin V, and flow cytometer was used for analysis. (**E**) MDA–MB-468 cells were treated as described in panel C, and terminal transferase mediated dUTP-fluorescein nick end labeling (TUNEL) staining was performed (**F–G**) MDA–MB-468 cells were treated with 10 μM of GL, SG-1709, and SG-1721 for various intervals, and Western blot analysis was done. (**H**) MDA–MB-468 cells were transiently transfected with STAT3 or scrambled si-RNA (control). Then, the transfected cells were treated with 10 μM of SG-1721 for 4 h. Thereafter, Western blot analysis was carried out. (**I**) The transfected MDA–MB-468 cells were treated with 10 μM of SG-1721 for 24 h, and then annexin V assays was carried out as described above in panel D.

Next, we proceeded to test whether GL and its analogues can also exert cytotoxic effects against TNBC MDA–MB-468 cells and breast epithelial MCF-10A cells. Interestingly, it was noted that the cell viability of tumor cells was reduced to the maximum level at 10 μM dose of SG-1721 among the others analyzed. By contrast, all had a relatively minor effect on the viability of normal cell line MCF-10A (Figure 3A). In addition, it was also observed that GL and its analogues significantly inhibited cell proliferation, and SG-1721 treatment inhibited cell proliferation at the highest rate (Figure 3B).

### 3.6. SG-1721 Can Induce Cell Cycle Arrest and Promote Apoptosis

We performed multiple biochemical assays to determine the effect of the SG-1721 on apoptosis. As depicted in Figure 3C, it was observed that cells accumulated to a greater extent in the S phase upon drug exposure. We next analyzed the effect of SG-1721 on annexin V binding and noted that the treatment caused the percentage of apoptotic cells to be higher than in nontreated cells, increasing from 2.5% to 12.6% (Figure 3D). The percentage of cells that underwent apoptosis also increased significantly after treatment with SG-1721, from 2.7% to 17.4%, as observed following TUNEL assay (Figure 3E). Further, as shown in Figure 3F, a substantial activation of caspase-3 and PARP cleavage was noted upon SG-1721 exposure. Additionally, Western blot analysis showed that SG-1721 can also markedly reduce the expression of Bcl-xL, Bcl-2, Cyclin D1, MMP-9, MMP-2, and COX-2 proteins (Figure 3G). We next tested whether SG-1721 induces cell apoptosis through inhibiting the STAT3 pathway, and transient transfections were carried out using STAT3 and scrambled si-RNA (control) in MDA–MB-468 cells. As shown in Figure 3H, phosphorylated and total STAT3 expression were substantially blocked upon transfection with STAT3/si-RNA. Subsequently, we observed that the knockdown of STAT3 with STAT3/si-RNA reversed the proapoptotic effects induced by SG-1721. The fold change in apoptotic cells was reduced from 8.5%:1.5% to 3.9%:1.7% (Figure 3I).

### 3.7. SG-1721 Can Sensitize TNBC Cells to Radiotherapy

**Figure 4:**
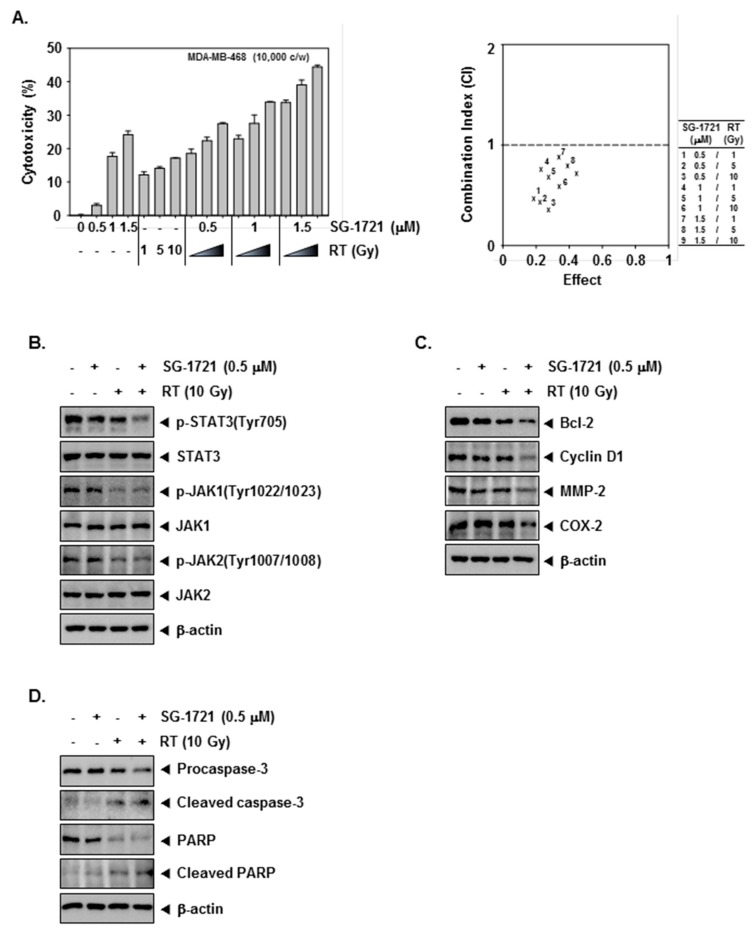
The effect of SG-1721 in combination with radiation on STAT3 signaling cascade (**A**) MDA–MB-468 cells treated with irradiation (1, 5, or 10 Gy) after 4 h of pretreatment with SG-1721 (0.5, 1, or 1.5 μM) incubated for 20 h. The cytotoxicity was determined by MTT assays (**left panel**). SG-1721 synergistically enhances radiation-induced cell death in MDA–MB-468 cells (**right panel**). The average of the CI values was obtained at nine different combinations. (**B**) MDA–MB-468 cells were treated with 0.5 μM of SG-1721 or 10 Gy of irradiation alone or in combination for 4 h. Thereafter, Western blot analysis was performed. (**C**) MDA–MB-468 cells were treated with irradiation (10 Gy) after 4 h of pretreatment with SG-1721 (0.5 μM) and incubated for 20 h followed by Western blot analysis. (**D**) MDA–MB-468 cells were treated as described above in panel C, and Western blot was performed using various antibodies.

The enhancement effect of SG-1721 on the radio-sensitivity of MDA–MB-468 cells was analyzed by MTT assay following treatment with various concentrations of SG-1721 (0.5, 1, or 1.5 μM) after irradiation with different doses of IR (1, 5, or 10 Gy). According to the results, the highest synergic cytotoxicity was obtained when the combined treatment of 0.5 μM of SG-1721 and 10 Gy of IR was used. (Figure 4A, left panel). The combination index (CI) indicated that effects are prominent at a combination dose of 0.5 μM of SG-1721/10 Gy of IR, which synergistically inhibited growth (Figure 4A, right panel). Moreover, as shown in Figure 4B, SG-1721 and IR (10 Gy) treatment alone showed minimal effects on the levels of p-STAT3 as well as p-JAK-1/2, while the combination treatment clearly suppressed activation of these oncogenic proteins in MDA–MB-468 cells. Further, as shown in Figure 4C, SG-1721 or IR (10 Gy) alone showed minimal effects the expression of Bcl-2, Cyclin D1, MMP-2, and COX-2 proteins. However, the combination treatment substantially reduced the expression of these proteins. We next investigated whether caspase activities are required to enhance SG-1721-mediated radiosensitive apoptosis. Combination treatment of SG-1721 and IR showed that cleavage of PARP and the expression of cleaved caspase-3 was found to be increased (Figure 4D). These results provide distinct evidence that SG-1721 can indeed sensitize TNBC cells exposed to radiation, and inhibition of p-STAT3 may play a key role in inducing this effect.

### 3.8. SG-1721 Can Inhibit Tumor Growth and Alter the Expression of Oncogenic Biomarkers In Vivo

**Figure 5:**
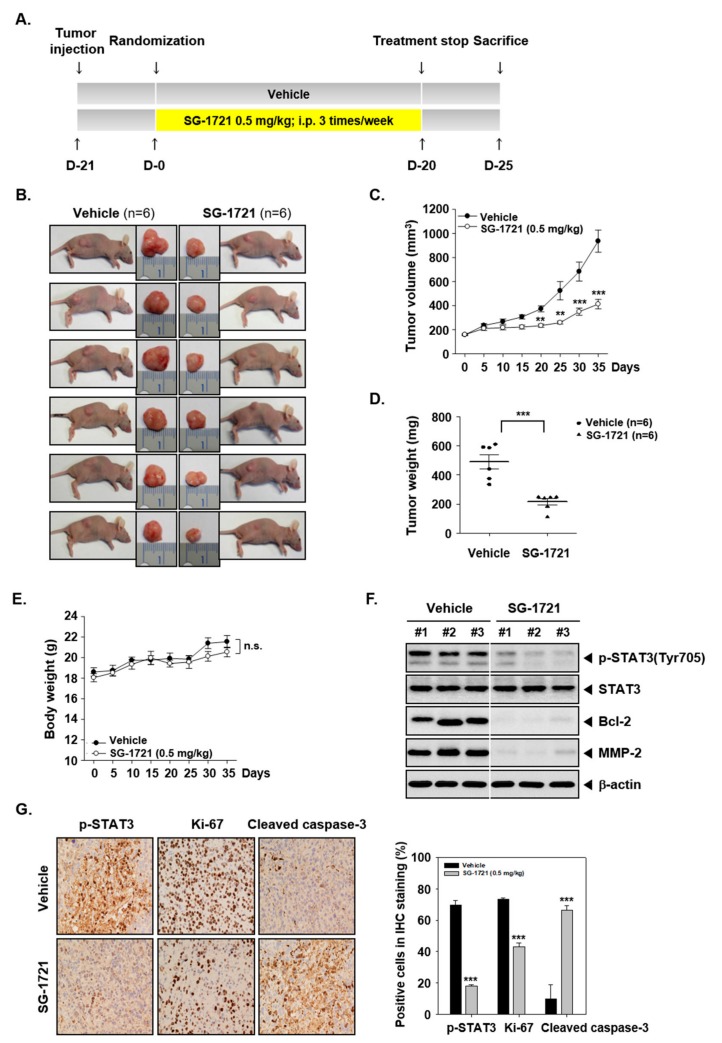
SG-1721 inhibits tumor growth in vivo. (**A**) A schematic representation of experimental protocol described in “Materials and Methods”. (**B**) Necropsy photographs of mice bearing subcutaneously implanted triple-negative breast cancer (TNBC) cells. (**C**) The tumor diameters were measured at 5-day intervals, and the tumor volumes were calculated using the formula V = 4/3 πr^3^ (*n* = 6). (**D**) Tumor weight was measured during the experiment. (**E**) Body weight changes of mice were measured at indicated times. (**F**) Western blot of various proteins of interest was carried out in lysate from vehicle control and SG-1721 treated mice. (**G**) Immunohistochemical analysis of p-STAT3, proliferation marker Ki-67, and cleaved caspase-3 in the tumor tissues (**left panel**). The results shown are representative of the three independent experiments. Graphs represent positive cells in IHC staining (**right panel**).

The preclinical effects of SG-1721 were analyzed as per the protocol depicted in Figure 5A. We noted that the treatment with SG-1721 resulted in a significant suppression of tumor size following drug exposure (Figure 5B). The tumor growth in the SG-1721 group was significantly more abrogated than in the vehicle group (Figure 5C,D) with an increase in the body weight of the mice (Figure 5E). We also noticed that SG-1721 was effective in reducing the expression of various proteins, including p-STAT3 in tumor tissues (Figure 5F). Interestingly, we further observed that SG-1721 substantially suppressed STAT3 phosphorylation, proliferation marker Ki-67, and increased cleaved caspase-3 levels in tumor tissues (Figure 5G), thus depicting the potent anticancer effects of SG-1721 in vivo. 

## 4. Discussion

The focus of this study was to analyze the activity of GL analogues against tumor growth in TNBC cell lines and a preclinical model. We observed that GL analogue can be more effective than GL in exerting their anticancer effects through targeted abrogation of the STAT3 mediated signal transduction cascade. 

We first measured STAT3 expression levels in several molecular subtypes of breast cancer cell lines and observed that STAT3 was phosphorylated persistently in TNBC cell lines. TNBC is an aggressive disease with poor prognosis because there are no specific treatment options for it. Thus, inhibition of phosphorylated STAT3 may be a potential strategy for the treatment of TNBC. We evaluated the effects of GL and GL analogues SG-1709 and SG-1721 on the STAT3 phosphorylation at Tyr705 residues using diverse assays. We also determined in detail the mechanisms by which GL and GL can negatively regulate STAT3 activation in TNBC cells. 

STAT3 is active constitutively in a variety of human cancer cells and tissues, including multiple myeloma and lung cancer [29,54]. It is considered to be an oncogene because it has the ability to promote malignant tumors [55]. STAT3 regulates the expression of various genes in response to cell stimulation and plays an important role in many cell processes, such as cell growth and apoptosis [56]. STAT3 is phosphorylated by JAK in response to ligands such as interferon, epidermal growth factor (EGF), IL-6, and translocation to cell nucleus acting as transcription activators [57]. In addition to direct activation of STAT3 protein by receptor tyrosine kinases, it can also be activated via upstream kinases such as JAKs [58]. The activity of JAK is induced when a regulatory molecule binds and two receptor molecules form a dimer [54]. We previously found that GL analogues SG-1709 and SG-1721 effectively inhibited the phosphorylation of STAT3 in a concentration- and time-dependent manner as compared to GL. SG-1709 and SG-1721 also abrogated JAK1 and JAK2 phosphorylation and inhibited IL-6 or EGF-induced STAT3 activation. 

Caspases are a group of protease enzymes that play an essential role in programmed cell death and inflammation [59]. Activation of caspases allows cell death to be controlled while minimizing the effect of cellular components on surrounding tissues [60]. We found that SG-1721 significantly increased cleavage of caspase-3 and PARP than GL. We demonstrated that SG-1721 inhibits the expression of various oncogenic proteins controlled by STAT3 activation. These results correlated with increased apoptosis, as demonstrated upon the drug exposure in TNBC cells. Previous reports have also shown that GL can affect different oncogenic signaling pathways in prostate cancer DU145 cells, thereby resulting in cell cycle arrest [61]. We also found that SG-1721 can induce cell cycle arrest at the S phase in TNBC cells.

Combination therapy can be used to reduce the likelihood of tumors becoming resistant to treatment and has the advantage of affecting multiple targets simultaneously during the treatment process [62]. We demonstrated the anticancer effect of low-dose SG-1721 and radiation combination therapy and identified a novel mechanism mediated by abrogation of STAT3 activation through which the SG-1721 analogue can sensitize TNBC cells to radiation. Finally, a significant anticancer effect of SG-1721 was demonstrated in breast cancer xenograft mouse models. Overall, SG-1721 has been conclusively found to significantly inhibit tumor growth without exhibiting any side effects and negatively regulate STAT3 activation in TNBC models.
